# Nanoplastic alters soybean microbiome across rhizocompartments level and symbiosis via flavonoid-mediated pathways

**DOI:** 10.3389/fpls.2025.1676933

**Published:** 2025-10-02

**Authors:** Muhammad Shoaib, Gen Li, Xinru Liu, Muhammad Arshad, Huijuan Zhang, Muhammad Asif, Marian Brestic, Milan Skalicky, Jun Wu, Shixiang Zhang, Feng Hu, Huixin Li

**Affiliations:** ^1^ The Sanya Institute of Nanjing Agricultural University, Nanjing Agricultural University, Sanya, China; ^2^ Soil Ecology Lab, College of Resources and Environmental Sciences, Nanjing Agricultural University, Nanjing, China; ^3^ College of Life Sciences, State Key Laboratory for Wheat Improvement, Shandong Agricultural University, Tai'an, China; ^4^ Institute of Plant and Environmental Sciences, Faculty of Agrobiology and Food Resources, Slovak University of Agriculture, Nitra, Slovakia; ^5^ Department of Botany and Plant Physiology, Faculty of Agrobiology, Food and Natural Resources, Czech University of Life Sciences, Prague, Czechia; ^6^ Jiangsu Provincial Key Laboratory of Coastal Saline Soil Resources Utilization and Ecological Conservation, Nanjing Agricultural University, Nanjing, China; ^7^ Zhengzhou Tobacco Research Institute of CNTC, Zhengzhou, China

**Keywords:** plastic pollution, nanoplastics (NPs), soil microbiome, soybean symbiosis, flavonoids biosynthesis, microbial assembly

## Abstract

Plastic pollution, particularly its breakdown into nanoplastics (NPs), poses a significant threat to ecosystem services, with notable effects on soil-plant-microbe interactions in agricultural systems. However, there is limited understanding of how NPs influence the soil microbiome and plant symbiotic functions. In this study, we applied polypropylene (PP) and polyethylene (PE) NPs, measuring 20 to 50 nm, to soybean growing conditions. We evaluated soil physicochemical properties, nodule counts, nitrogenase activity, and bacterial community composition in nodule, rhizosphere, and bulk soil under different concentrations of these NPs (200, 500, and 1000 mg/kg of soil w/w). Our results revealed that the impact of NPs on soil physicochemical properties was type-dependent, with PE-NPs exerting a more pronounced effect on soil enzyme activities than PP-NPs. Both NPs treatments accelerated nodulation and increased nitrogenase activity, with lower doses inducing more significant effects. Furthermore, PE and PP-NPs enriched bacterial species such as *Ensifer* and *Arthrobacter*, which positively interact with diazotrophs such as *Bradyrhizobium*, supporting symbiosis and biological nitrogen fixation. NPs treatments also significantly affected the bacteriome assembly process in the bulk soil, rhizosphere, and nodule, with an increased source ratio from the rhizosphere to the nodule and homogenous selection in the nodule bacteriome, likely benefiting bacteria involved in nodulation. Exposure to 500 mg/kg of both NPs caused alterations in the metabolic exudation profile of the plant rhizosphere, particularly influencing the biosynthesis pathways of flavonoids and isoflavonoids. Metabolites such as genistein and naringenin emerged as key mediators of plant-microbe interactions, further enhancing plant symbiotic processes under NPs exposure. This study demonstrates that NPs influence plants’ symbiotic potential both directly, by altering the composition of the soil bacteriome, and indirectly, by affecting exudation potential. It provides strong evidence that NPs, especially those smaller than a micrometer, can have long-term effects on the stability and functionality of agricultural ecosystems.

## 1 Introduction

Since the 1950s, global plastic consumption and production have increased by 4% annually (Bratovcic, 2024). This growth has outpaced waste management capacity, with only 16% of plastic products being recycled, while 66% are discarded, resulting in widespread environmental pollution and serious ecological and agricultural concerns (Nicholson et al., 2021). Agricultural soils, subjected to intensive anthropogenic activities, are a significant source of plastic pollution due to inputs such as plastic film (He et al., 2023), polymer-containing fertilizers, biosolids, compost (Vithanage et al., 2021), sewage sludge, wastewater irrigation (Pérez et al., 2022), and atmospheric deposition (Brahney et al., 2020). Plastic pollution in soils can range from 0.34 to 410958.90 items/kg, and concentration ranged from 0.00 to 67500.00 mg/kg, across sites (Wei et al., 2022). Advanced analytical methods have detected submicron plastics in the soil area studied. Polypropylene (PP) accounted for the most significant proportion (up to 34%); the particle size of the obtained microplastics ranged from 0.50 to 25 μm, with over 59% of the plastic particles being <15 μm in size (Du et al., 2020). As a subset of microplastics (MPs, <5mm), (Nanoplastics; NPs <5μm) have become a particular concern due to their smaller size, greater mobility, and potential for cellular internalization and subsequent toxicological effects (Khanna et al., 2021; Rose et al., 2023). For instance, polyvinyl chloride (PVC) NPs were shown to be more readily absorbed by rice roots and subsequently translocated to the shoots, leading to greater nutritional impairment and oxidative damage than PVC-MPs (Galahitigama et al., 2024). Similarly, the activity and diversity of microbial communities in sludge were more significantly inhibited by exposure to 213nm polystyrene (PS) particles compared to larger 633µm and 3.3mm PS treatments (Xu et al., 2021). Although the effects of NPs on agricultural soils are less studied than those of larger plastics, limited data exist on how NPs influence soil microbial community composition, assembly processes, and ecosystem functioning, particularly through their impacts on plant-soil-microbe interactions.

Due to their distinct composition and hydrophobic surfaces, NPs differ significantly from natural soil particles, allowing them to alter soil properties such as pH, nutrient availability, water retention, porosity, and bulk density (Ding et al., 2022; Wang et al., 2021; Zhou et al., 2021). These changes can stimulate or inhibit soil enzyme activities, which are key drivers of biogeochemical cycles and indicators of microbial functionality (Song et al., 2025). In addition to modifying soil chemistry, plastics can serve as new carbon sources and provide habitats for microbes, thus fostering unique microbial communities that diverge from surrounding soil (Liu et al., 2025). Studies on various polymers, including PVC, polyurethane foam (PUF), polylactic acid (PLA), and PS, have revealed alterations in the microbial communities in sediments (Giroux et al., 2023; Huang et al., 2021). However, results remain inconsistent, underscoring the influence of particle size, polymer type, concentration, and soil properties (Graf et al., 2023). Although shifts in microbial composition are increasingly reported, understanding the underlying assembly mechanisms, whether deterministic or stochastic, provides deeper insights into ecosystem functioning (Chen et al., 2021; Li et al., 2025). Recent findings have demonstrated that NPs disrupt symbiotic relationships with legumes, altering nitrogen fixation and cycling (Ya et al., 2021; Zhai et al., 2024). This impact is multifaceted; for example, NPs may restrict microbial mobility, which hinders the establishment and maintenance of beneficial mutualistic relationships. NPs can sequester essential nutrients, thus diminishing their bioavailability to rhizobia, leading to perturbations in the dynamics of the microbial community and ultimately affecting the symbiotic potential (Wang et al., 2023). For example, high polyethylene (PE) and rubber crumbs concentrations repressed peanut development and nitrogen absorption by detrimental root cells, disrupting the soil nitrogen cycle (Liu et al., 2023). On the contrary, certain nanosized plastics, such as PVC and PS, have been found to positively affect legume symbiotic efficiency and the microbiome of the rhizosphere by altering soil enzyme activity and modifying diazotroph communities (Shah et al., 2023).

Bacterial colonization or growth in the nodules or roots of legume plants is influenced by bacterial chemotaxis toward root exudates over short distances or through other means, like mycelial networks (Zhang et al., 2020). Early rhizobia colonization is crucial for effective nodulation and efficient nitrogen fixation, supporting rapid plant growth (Boyle et al., 2021). Abiotic factors selectively influence symbionts, shaping microbial communities by allocating resources to roots and nodules, leading to changes in rhizobia diversity (Dinnage et al., 2019). Root exudates play a crucial role in modulating the microbial dynamics of the rhizosphere, facilitating the solubilization of nutrients and nitrogen fixation. These exudates include organic acids, fatty acids, and stress-responsive metabolites (Bouaicha et al., 2022) and contain specialized compounds such as flavonoids that help orchestrate the rhizobium-legume symbiosis by selectively recruiting rhizobia (Singla and Garg, 2017). However, the mechanisms by which NPs influence rhizosphere exudation and affect symbiotic potential in legumes remain poorly understood. We hypothesize that (i) NPs may alter nutrient and enzymatic balance, (ii) NPs influence legume symbiotic potential by altering the metabolic profile of plant exudates, (iii) NPs affect microbial community assembly process and composition across different rhizocompartments, and (iv) NPs may promote microbial exchange via the plastisphere or inhibit microbial movement by blocking soil pore connectivity. To test these hypotheses, we used soybean (*Glycine max* cv. Nandou 12), a model legume, and PE and PP as representative nanosized plastics. PE and PP are the two most common plastics found in soil, and the World Health Organization has classified PE as a Class 3 carcinogen, highlighting the potential long-term risks associated with plastic pollution in the environment (Teng et al., 2022).

## 2 Materials and methods

### 2.1 Material

The surface soil (0–20 cm) was collected from the agricultural soil at the Baima Teaching and Research Base (31°36′56″N; 119°10′31″E). The site’s sandy loam soil has a well-documented history, and no plastic film has been applied; furthermore, there has been no recorded plastic contamination. Basic soil properties are detailed in the Supplementary Material (Section 1.2). Before use, we meticulously removed the soil from visible plant debris. After air-drying, the soil was sieved through a 2 mm mesh for consistency. We selected *Glycine max* cv. Nandou 12 seeds (soybean) for the experiment. The PE and PP-NPs, measuring 20 to 50 nm in diameter, were sourced from Jiangsu Zhongfu New Materials Co., Ltd. in China. We used scanning electron microscopy (SEM) with the HITACHI SU8600 and JW-BK200C models from Gaobo, China, to examine the morphological characteristics of the plastics, confirming their particle size and surface structure (Supplementary Materials and Methods, Section 1.3).

### 2.2 Experimental design

A pot experiment was conducted in the growth room of Nanjing Agricultural University in Nanjing, China, to investigate the effects of various types of NPs on plant growth and soil microbiome. The experiment took place from April to November 2023 and employed a randomized block design with two factors: plant and NPs. In total, seven treatments were involved: CK (no NPs), PP1 (200 mg/kg of PP), PE1 (200 mg/kg of PE), PP2 (500 mg/kg of PP), PE2 (500 mg/kg of PE), PP3 (1000 mg/kg of PP) and PE3 (1000 mg/kg of PE). The plastic concentration used in this experiment was selected based on findings from previous studies (de Souza et al., 2019). The plastics were thoroughly mixed with the soil, and 50 grams of nutrient-rich soil was added to each pot as the base fertilizer. We surface sterilized the soybean seeds to eliminate potential contaminants and germinated them in trays. After one week, the seedlings were transferred to the pots. We used five replicates for each treatment, with one seedling per pot. Subsequently, we placed these pots in a controlled environment chamber, including light intensity of (400 μmolm^-2^s^-1^) and a photoperiod of 16/8 hours of light/dark, a day/night temperature of 25/20°C, and watered regularly to keep the soil moist.

### 2.3 Plant and soil sampling

After 40 days of growth, nodules, rhizosphere and bulk soil samples were collected, following the method outlined by Bulgarelli et al. (2012). After harvesting, we quantified soybean biomass using the drying and weighing protocol. Root samples were immediately stored -80°C after harvesting for physiochemical analysis. To assess the symbiotic effectiveness of soil microbes under specific conditions, the number of nodules was manually counted. Also, nodules samples were collected to analyze nitrogenase activity and for subsequent DNA extraction/sequencing. One hundred and five samples were collected for the microbial community study, consisting of 35 bulk soil, 35 rhizosphere, and 35 nodule samples. These samples were taken with 5 biological replicates in 7 treatments for each niche compartment. To collect the soil samples, the soil from each pot was homogenized to create a representative mixture to analyze the nutrient content and enzyme activity. All analyses at the plant and soil level were performed using five biological replicates. A detailed protocol for NPs accumulation measurement in roots, soil, and plant physicochemical analysis is provided in the supplementary materials and methodology section (1.4, 1.5, and 1.6).

### 2.4 Rhizosphere soil metabolomic profile

Rhizosphere soil collection and metabolomic analysis were conducted using a detailed methodology previously explained by Guo et al. (2020). The roots were harvested, and the loose soil was removed by shaking and kneading while wearing sterilized gloves. The soil adhered to the roots, which were carefully brushed off and then frozen in liquid nitrogen. The samples for metabolomic analysis were extracted using a solution of 80% methanol (v/v) with 0.1% formic acid, followed by centrifugation at 15,000 × g for 10 minutes at 4°C. The resulting supernatants were then analyzed. Only two treatments, PP2 and PE2, were selected for metabolomic analysis due to their significant effects on various parameters, including nodulation and nitrogen fixation. HPLC-MS/MS analyses were performed using a Vanquish UHPLC system (Thermo Fisher, Germany) coupled with an Orbitrap Q ExactiveTM HF mass spectrometer (Thermo Fisher, Germany) at Biozeron Co., Ltd. (Shanghai, China). Peak signal intensities (peak areas) were selected and normalized in parts, applying a standard threshold relative standard deviation (RSD) of <0.3. Redundancy and peak merging techniques were used to derive metabolite expression data (Zhao et al., 2019). Principal component analysis (PCA) and orthogonal partial least squares discriminant analysis (OPLS-DA) were performed after mean centering and unit variance scaling to visualize metabolic differences among the experimental groups. Metabolites with a variable importance in projection (VIP) value greater than 1 and a P-value of less than 0.05 (two-tailed Student’s t-test) were identified as differentially expressed metabolites (DEM). Comparisons were made between PP2 vs. control and PE2 vs. control. Metabolites were annotated using the Kyoto Encyclopedia of Genes and Genomes (KEGG) database (https://www.kegg.jp/), and pathway enrichment analysis was performed to visualize the top 20 pathways in bubble charts (Wolthuis et al., 2020).

### 2.5 DNA extraction and 16S rDNA sequencing

Genomic DNA was extracted from bulk and rhizosphere soil using the PowerSoil^®^ DNA Isolation Kit (MoBio Laboratories, Carlsbad, CA). The nodules were collected, surface sterilized with 70% ethanol, frozen, and ground. DNA from the nodules was extracted using the DNeasy Plant Mini Kit (QIAGEN). A NanoDrop One Spectrophotometer (Thermo Scientific, Wilmington, USA) was used to assess the quality and concentration of the extracted DNA. The V4 and V5 regions of 16S rDNA were amplified using universal primers 515 F (5’-GTGCCAGCMGCCGCGGTAA-3’) and 907 R (5’-CCGTCAATTCCTTTGAGTTT-3’) (Jing et al., 2015; Sun et al., 2015). The PCR products were sequenced on the Illumina MiSeq platform (Illumina, USA). Sequencing data were analyzed using the quantitative insights into microbial ecology pipeline (QIIME2-2020.11) (https://docs.qiime2.org/2020.11/tutorials/) (Bolyen et al., 2019). First, the raw data were spliced and filtered to generate clean reads, followed by noise reduction using DADA2 to produce the final amplicon sequence variants (ASV). We annotated the species for each ASV using the SILVA 138.1 database and constructed an ASV table. Raw sequence data were stored in the NCBI Sequence Read Archive: PRJNA1156063, PRJNA1156377, and PRJNA1156528.

### 2.6 Data analysis

We performed a complete two-way ANOVA using Statistix 8.1 to evaluate the influence of NPs on plant performance and soil properties. The investigation comprised two fixed factors: plastic type (PE and PP) and concentration (100, 200, and 1000 mg/kg of soil). This strategy enabled evaluating both primary effects and potential interactions between the two components. We evaluated alpha diversity (species richness and evenness) using the Shannon index, Pielou’s evenness, and the Chao1 index. We evaluated beta diversity *via* nonmetric multidimensional scaling (NMDS) and unweighted UniFrac distance to examine compositional variations across treated groups. Using the Mantel test, we correlated Euclidean distances of soil properties with the top ten bacterial communities at the genus level. We estimate microbiome sources across ecological niches with fast expectation-maximization microbial source tracking (FEAST). To analyze and quantify the mechanisms behind the assembly of the microbial community, various community assembly processes, homogeneous selection (HoS), heterogeneous selection (HeS), dispersal limitation (DL), and drift (DR), were identified using the iCAMP package, based on a previous study (Ning et al., 2020). We performed data visualization using Origin and R packages “vegan” and “ggcor” (v.4.1.2) (Oksanen et al., 2018).

## 3 Results

### 3.1 Changes in plant growth, nodulation, and nitrogenase activity

**Figure 1:**
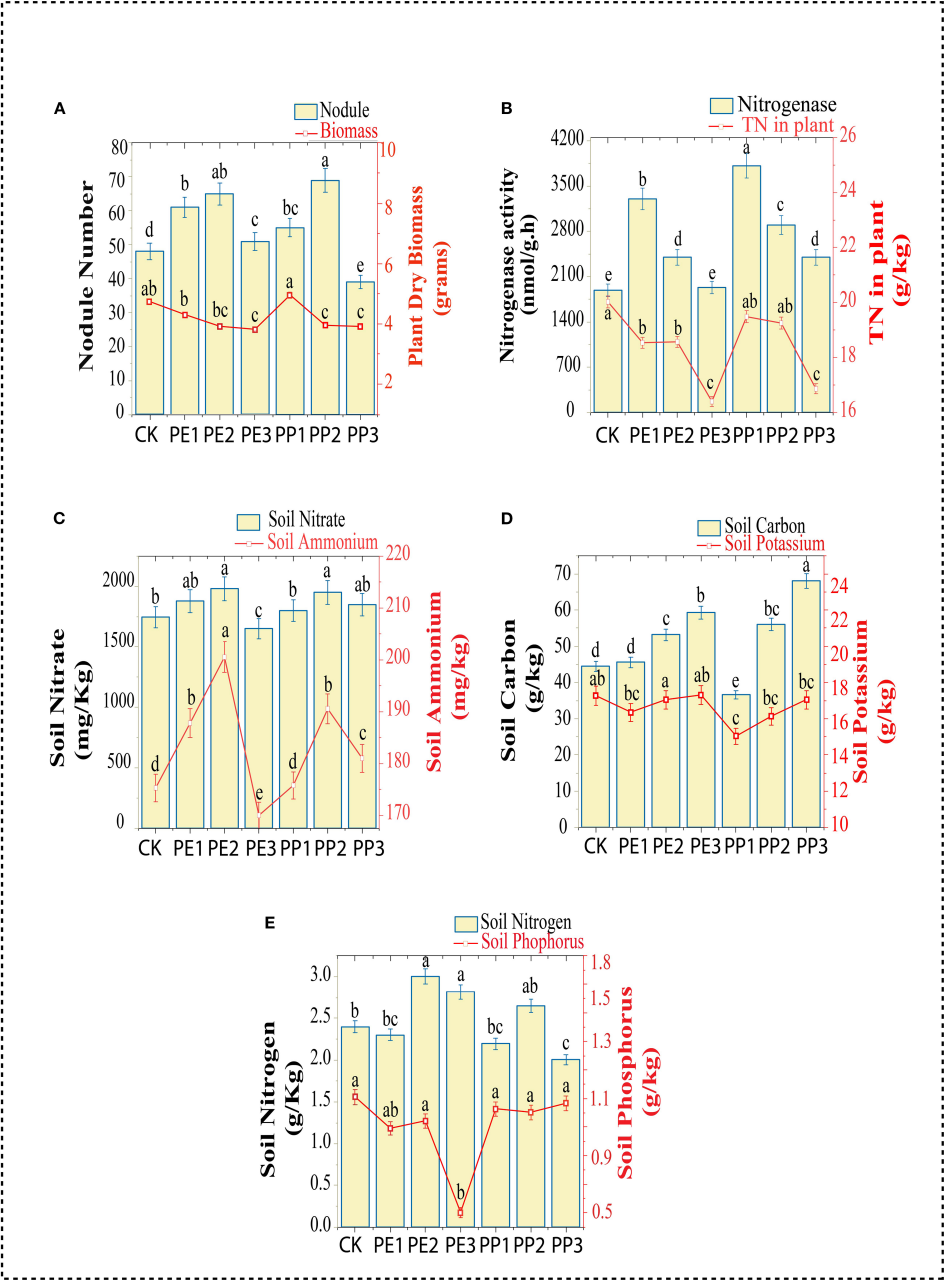
Changes in Plant **(A)** nodule number, biomass, **(B)** Nodule nitrogenase activity, total nitrogen content in plant, **(C)** Soil nitrate and ammonium content, **(D)** Soil total carbon and potassium content, **(E)** Soil total nitrogen and phosphorus content. CK indicates control with no plastic particles added, and PE1, PE2 and PE3 represents the treatments with 200, 500 and 1000 mg/kg of polyethene, respectively. While PP1, PP2 and PP3 correspond to treatments with 200, 500 and 1000 mg/kg of polypropylene. All NPs were added exogenously to the soil. Values (mean ± SD, n = 5) with different superscripts differ significantly (Two-way ANOVA, Tukey’s HSD, p < 0.05).

The total dry biomass of soybean was reduced in all treatments except PP1; however, the reduction was particularly evident in the PE3 and PP3 treatment groups, which showed a significant decrease of 17 to 19% in dry plant biomass compared to control (**Figure 1A**). Plant nodulation improved with the first two doses of both NPs treatments, with increases of 25% to 43%, respectively, compared to the control (**Supplementary Figure S1**, phenotypic assessment). The nitrogenase activity of the nodules increased in all treatments compared to the control, with the PP1 and PE1 treatments showing the highest activity. The TN content in the plant decreased significantly under the PE3 and PP3 treatments (**Figure 1B**).

### 3.2 NPs accumulation in roots and antioxidant defense response

A significant (P < 0.05) accumulation of NPs was observed in the roots of soybean in all NPs-treated groups, compared to the control group, which showed no detectable NPs. PE-NPs accumulation exhibited a dose-dependent response, with higher doses (PE2 and PE3) resulting in a 5–7 fold increase in root PE accumulation compared to the control. In contrast, PP-NPs uptake was significantly (P < 0.05) lower than PE-NPs and remained unchanged with increasing NPs concentrations. Malondialdehyde (MDA) levels in the roots, used as a stress marker, also increased in a dose-dependent manner with the NPs concentration. Interestingly, despite lower accumulation of PP-NPs compared to PE-NPs, exposure to PP-NPs led to higher MDA content. The level of superoxide dismutase (SOD) enzyme activity increased in all NPs treatments, except for PP3, which caused a 6% decline compared to the control. Catalase (CAT) enzyme activity improved at lower concentrations of both types of NPs. However, at higher concentrations (500 and 1000 mg kg^−1^) of both NPs, CAT activity in soybean roots was found to decrease (**Supplementary Figure S2**).

### 3.3 Changes in soil physicochemical properties

**Figure 2:**
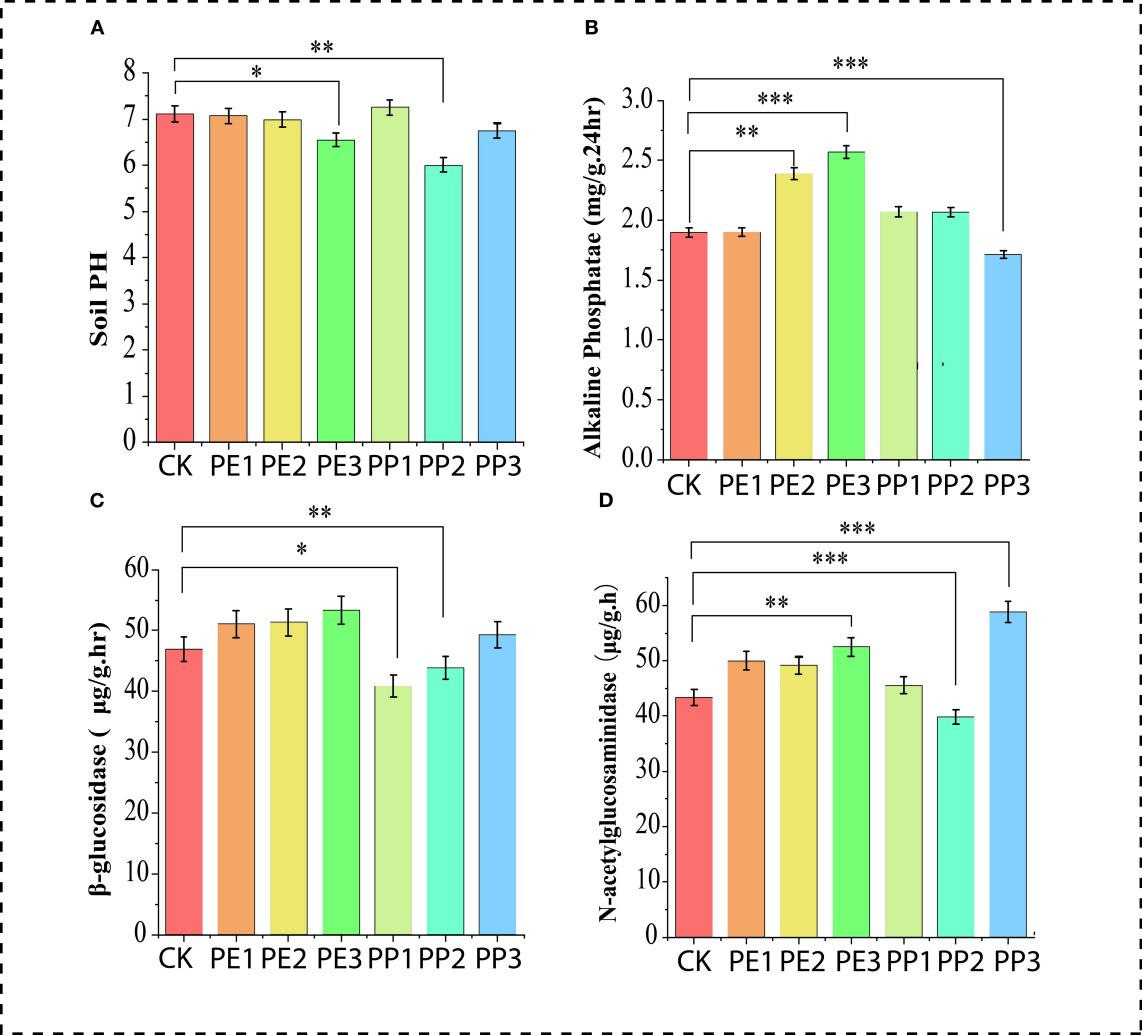
Changes in soil **(A)** pH, **(B)** alkaline phosphatase, **(C)** β-1,4-glucosidase, **(D)** N-acetyl-β-glycosaminidase activities with NPs exposure. Note: CK indicates control with no plastic particles added, and PE1, PE2 and PE3 represents the treatments with 200, 500 and 1000 mg/kg of polyethene, respectively. While PP1, PP2 and PP3 correspond to treatments with 200, 500 and 1000 mg/kg of polypropylene. All NPs were added exogenously to the soil. Values (mean ± SD, n = 5) with different asterisks (***,**,*) differ significantly (Two-way ANOVA, Tukey’s HSD, p < 0.05).

To gain critical insight into changes in soil fertility and nutrient availability induced by NPs in the soil, key macronutrients, including soil Ammonium (NH_4_^+^), Nitrate (NO_3_^–^), total nitrogen (TN), total organic carbon (TOC), total phosphorus (TP), and total potassium (TK) content were measured. For soil NH_4_^+^, lower values were detected at the highest doses of both NPs treatments, while the PE2 and PP2 treatments elevated these values compared to the control. There was no discernible difference between the control treatment and the PE3 and PP1 treatments, all other treatments showed an increase in the NO3 content of the soil (**Figure 1C**). The soil TOC content was consistently higher (P< 0.05) in most treatment groups relative to the control, with the most pronounced increase in the treatments (PE3 and PP3), showing elevations of 33% and 34%, respectively. No considerable variance was detected between the control and all NPs treatment groups in soil TK (**Figure 1D**). Concerning soil TN (PE2 and PP2), the treatments increased the TN content in the soil, although PP3 decreased the TN. Furthermore, soil from PE3 treatment had a lower concentration of TP relative to the control; other treatments had no significant impact on the level of phosphorus (**Figure 1E**). The effect of NPs on soil nutrients observed in our study is inconsistent with previous findings. These discrepancies can be attributed to plastic type, size, concentration, and growth conditions (Lan et al., 2025). Treatments with PP2 and PE3 reduced soil pH, while PP1 treatment improved it by 4%. However, no significant variation was observed in other treatments compared to the control (**Figure 2A**). Treatment with PE2 and PE3 increased soil alkaline phosphatase (AlP) activity, PP3 reduced it compared to the control (**Figure 2B**). In general, PP-NPs treatment reduced β-1,4-glucosidase (BG) activity. In contrast, PE-NPs enhanced it relative to the control. Regarding N-acetyl-β-glycosaminidase (NAG) activity, the treatments with PE3 and PP3 promoted its activity, with increases of 47% and 31%, respectively, relative to the control (**Figure 2C**). Overall, PE-NPs exhibit a more pronounced constructive influence on soil enzyme activities than PP-NPs, which tends to exert a more detrimental effect on soil enzyme activities.

### 3.4 Changes in the microbial community

**Figure 3:**
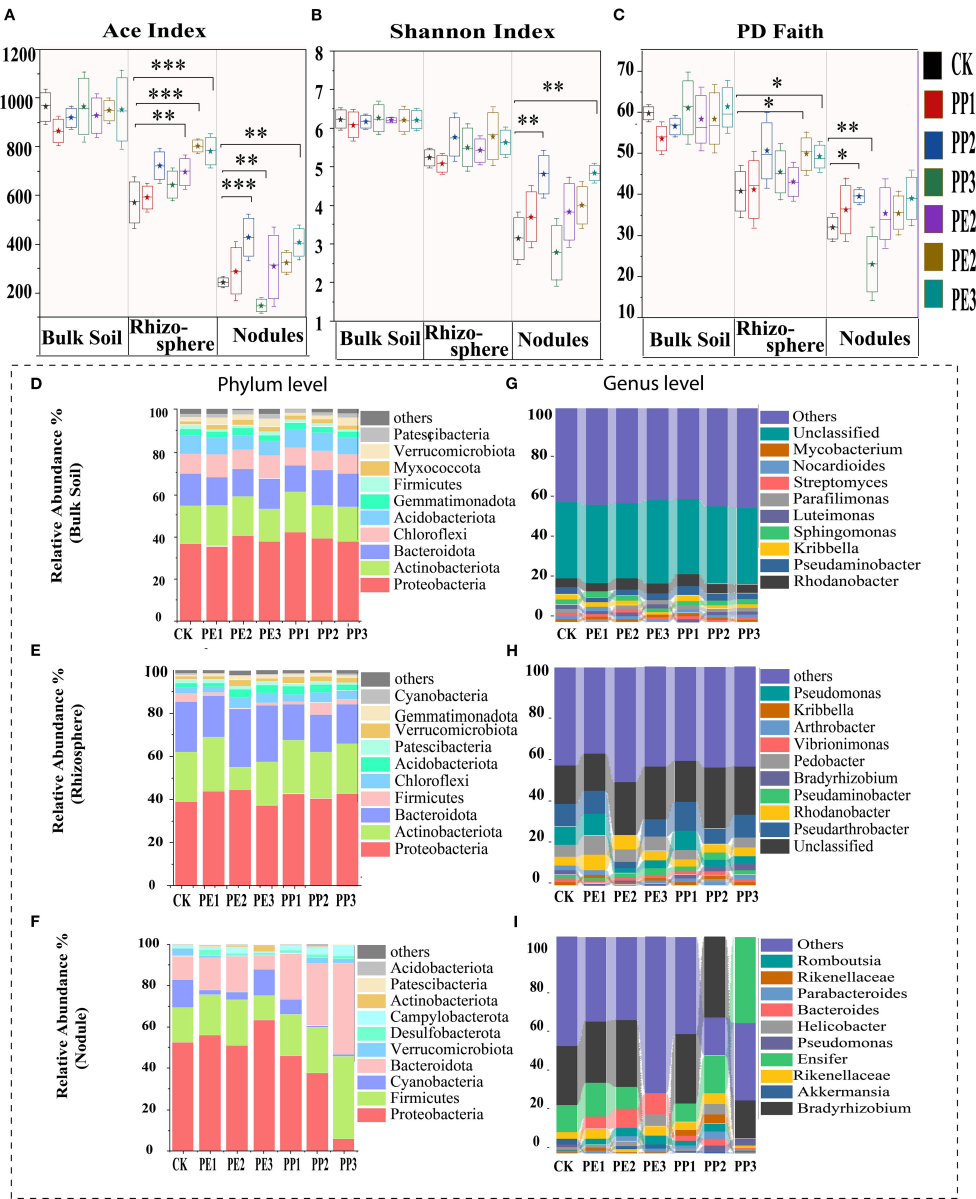
Changes in **(A)** ACE index, **(B)** Shannon, **(C)** PD faith, **(D)** and **(D–F)** relative abundance of bacterial community at phylum level and **(G–I)** at genus level within different rhizocompartments(Bulk Soil, Rhizosphere, Rooot Nodules) with varying concentration of NPs. Note: CK indicates control with no plastic particles added, and PE1, PE2 and PE3 represents the treatments with 200, 500 and 1000 mg/kg of polyethene, respectively. While PP1, PP2 and PP3 correspond to treatments with 200, 500 and 1000 mg/kg of polypropylene. All NPs were added exogenously to the soil. According to the T-test, with different asterisks (***,**,*), the results differ significantly (*P* < 0.05, n = 5).

Most NPs treated groups showed increased bacterial diversity in the rhizosphere and plant nodules compared to the control group, according to the alpha diversity measured by the Chao1, Shannon, and Faith PD indices. The most pronounced increase in diversity was observed in the treatment groups (PE3, PP3), followed by the treatment groups (PE2, PP2) (**Figure 3A**). Furthermore, the rhizosphere showed a significant increase in richness (Chao1) and evenness (Pd faith) in response to most NPs treatment groups (P< 0.05). Nodules exhibited a similar trend in richness and evenness, except under PE3 treatment (**Figures 3B, C**). While alpha diversity metrics reflected within-sample changes, NMDS further revealed pronounced shifts in microbial community composition across treatment groups, particularly in the rhizosphere and nodules (**Supplementary Figure S3**). In particular, alpha diversity was observed in the following sequence: bulk soil, rhizosphere, and nodule. This pattern probably reflects the reduced complexity of the microbial network as one transitions from bulk soil to more specialized endospheric compartments (rhizosphere and nodules) (Cregger et al., 2021).

In bulk soil, the phyla *Proteobacteria, Actinobacteria, Bacteroidota*, *Chloroflexi*, and *Acidobacteriota* represented the majority of annotated reads. They showed slight enrichment with mixed response under NPs treatment compared to the control (**Figure 3D**). Similarly, the rhizosphere microbiota was dominated by phyla analogous to those of bulk soil, and their relative abundance displayed treatment-specific variation. For instance, the addition of NPs consistently enriched *Bacteroidota*, *Proteobacteria* however showed mixed responses under different treatments (**Figure 3E**). *Firmicutes* and *Bacteroidota* were found highly enriched under different NPs treatments in nodules (**Figure 3F**). At the genus level, the composition of the microbial community in the bulk soil under both control and NPs treatments revealed striking similarities, with the unclassified genera *Rhodanobacter, Pseudaminobacter, Kribbella, Sphingomonas* and *Luteimonas* as the predominant taxa (**Figure 3G**). However, within the rhizosphere, unclassified genera exhibited a pronounced increase in relative abundance, further amplified under NPs treatments as depicted in (**Figure 3H**). In particular, the relative abundance of *Pseudarthrobacter* experienced a decline under PP2 sand PE3 treatments, with a reduction of 3% and 7% compared to the control. The relative abundance of *Bradyrhizobium* in the nodules displayed a nuanced response, increasing by 6% and 11% under PP1 and PP2, respectively, while decreasing by 10% and 27% with PP3 and PE3 amendments. On the contrary, the relative abundance of *Ensifer* exhibited an inverse pattern, declining by 12% under PE3 but increasing by 20% and 28% under the PP2 and PP3 treatments, respectively (**Figure 3I**). Similarly, Bacteroidetes demonstrated divergent trends across NPs treatments, highlighting the complex and treatment-specific dynamics of microbial communities.

### 3.5 Microbial dynamic assembly processes and transfer across the rhizocompartments level

**Figure 4:**
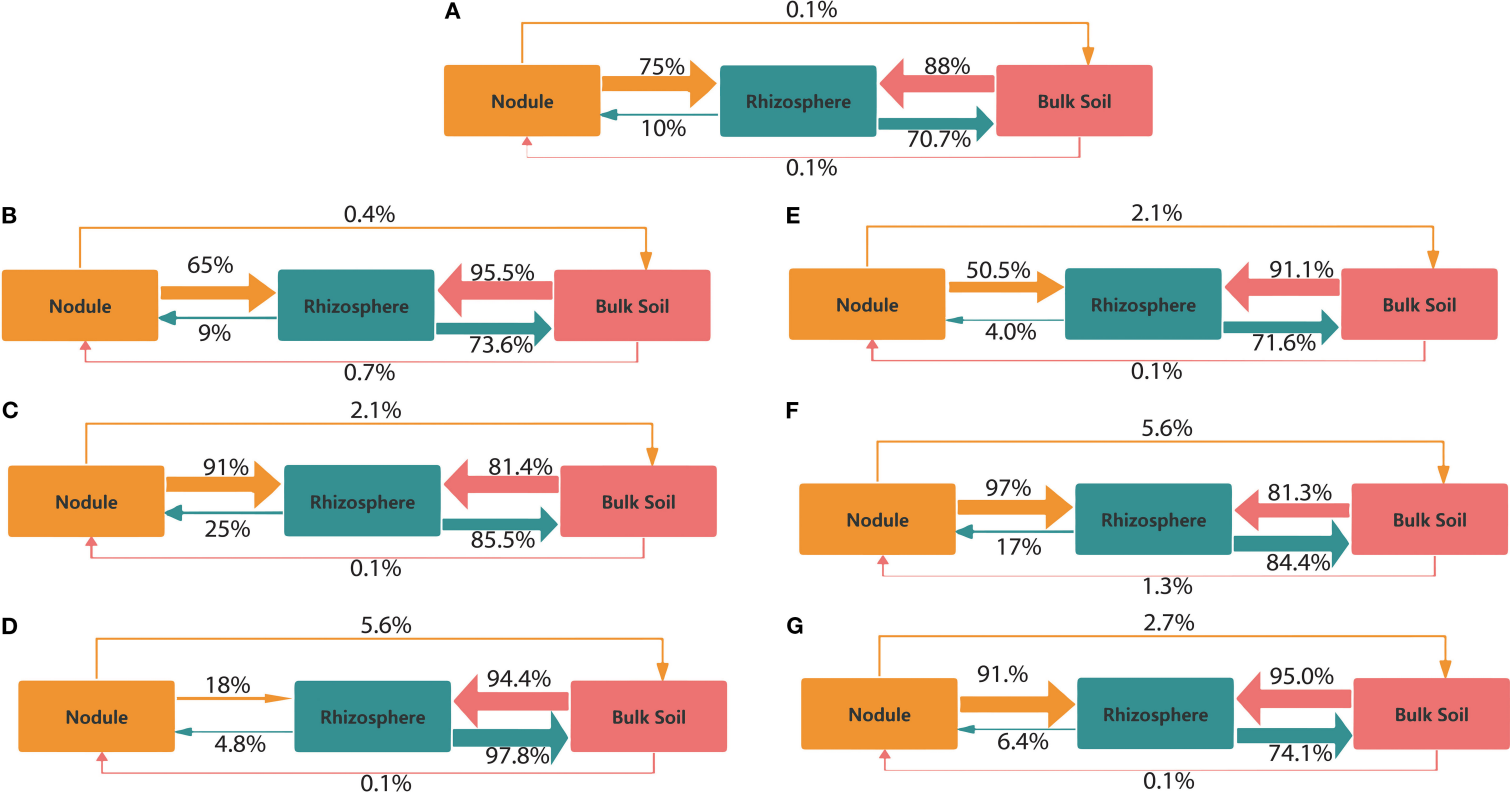
Source tracking for each pair of compartments under the following treatments: **(A)** CK, **(B)** PE1, **(C)** PE2, **(D)** PE3, **(E)** PP1, **(F)** PP2, and **(G)** PP3. While the arrows’ thickness represents their proportionate size, their orientation denotes the possible transmission path of microbes. Note: CK indicates control with no plastic particles added, and PE1, PE2 and PE3 represents the treatments with 200, 500 and 1000 mg/kg of polyethene, respectively. While PP1, PP2 and PP3 correspond to treatments with 200, 500 and 1000 mg/kg of polypropylene. All NPs were added exogenously to the soil.

FEAST analysis showed similarities within each pair of niche compartments. Across all NPs treatments, the transfer from the nodule to the rhizosphere ranged from 18% to 97%. The transfer bottleneck from the nodule to the rhizosphere was 18% under PE3 treatment (**Figure 4**). In contrast, the highest transfer was observed in the PE2 and PP2-treated group. Transfer from the rhizosphere to the nodule increased by 15% with PE2 treatment and by 7% with PP2 treatment, compared to the control. Transfers between bulk soil and rhizosphere consistently rose from 71% to 97% as NPs concentration ranged from lower to higher doses, compared to the control (**Figure 4**). Transfer between the nodule and the bulk soil ranged from 0.10% to 5.60% in all treatments, with a higher transfer observed from the nodule to the soil than from the soil to the nodule. All NPs treatments increased the contribution of the bulk soil community to the rhizosphere microbiome. In contrast, the PE2 and PP2 treatments boosted the relative contribution of the rhizosphere community to nodules.

**Figure 5:**
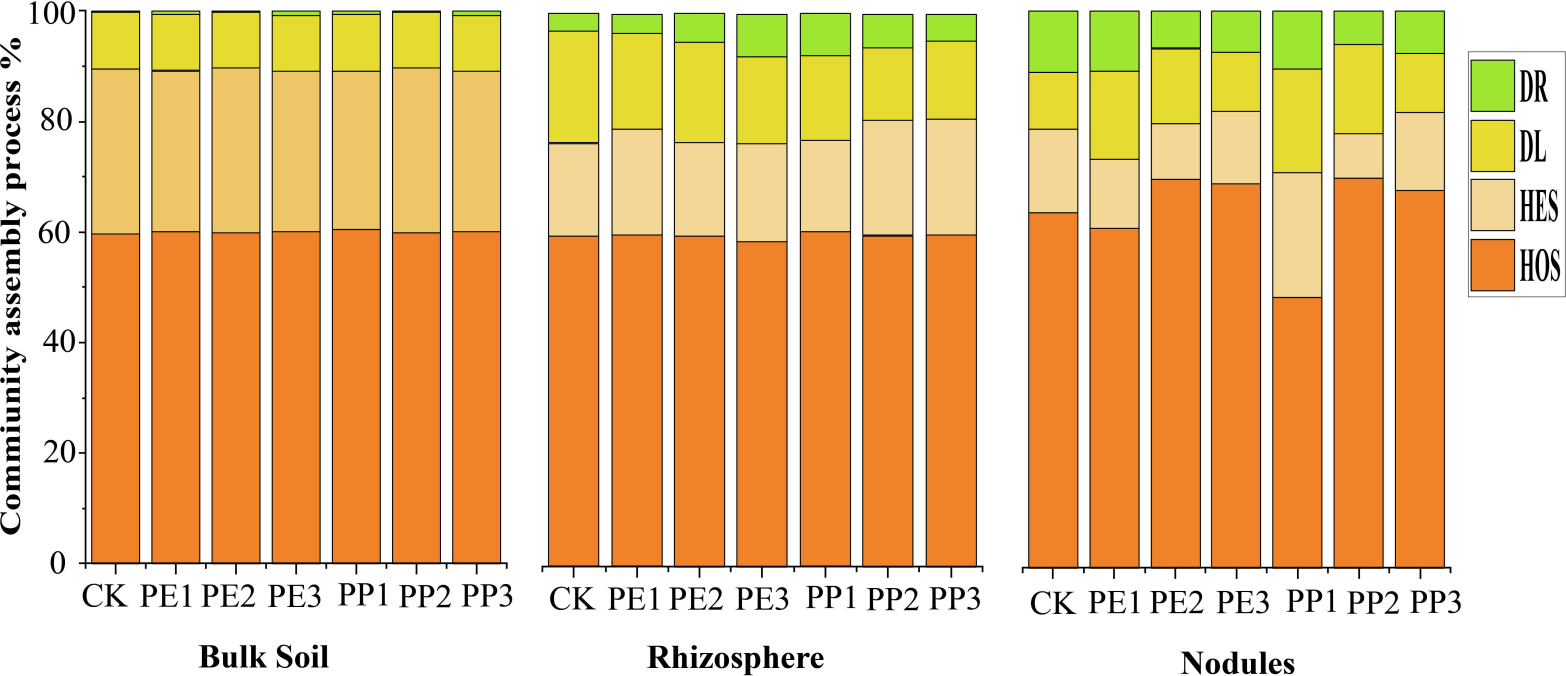
The relative position of different ecological processes in bacterial community assembly in different rhizocompartments levels. HoS, homogeneous selection; HeS, heterogeneous selection; DL, dispersal limitation; DR, drift. Note: CK indicates control with no plastic particles added, and PE1, PE2 and PE3 represents the treatments with 200, 500 and 1000 mg/kg of polyethene, respectively. While PP1, PP2 and PP3 correspond to treatments with 200, 500 and 1000 mg/kg of polypropylene. All NPs were added exogenously to the soil.

At all levels of rhizocompartments, the percentage of HoS contributed mainly to the microbial assembly processes, accounting for 60% to 70% of the bacterial community assembly processes. Under NPs exposure, heterogeneous selection HeS increased from 16% to 21%, and dispersal limitation DL decreased from 2% to 6% in the assembly of the rhizosphere community (**Figure 5**). In particular, the impact of PP3 treatment on the assembly of the bacterial community in the rhizosphere was relatively strong. HoS increased from 60.93% to 70.46% in nodule community assembly processes, and HeS decreased from 15.70% to 8% under NPs treatments. However, PP1 treatment increased HeS (from 15% to 22.40%) and decreased HoS from 63.83% to 48.41%. The impact of PE-NPs treatment on the bacterial community assembly was minimal. On the contrary, the PP1 treatment resulted in a slightly greater dispersal limitation (DL) contribution to the nodule assembly process, accounting for 7.48%. In the assembly of the microbial community of the nodules, the stochastic processes (DL + DR) over all increased under NPs treatments; however, differences were observed in type and dose for the processes of the microbial community (**Figure 5**). In bulk soil, both deterministic and stochastic processes contributed equally to community assembly in NPs treatments. A greater variation in stochastic processes was observed in the microbial community assembly of the rhizosphere and nodules under different NPs treatments.

### 3.6 Metabolomic profile of rhizosphere exudates

**Figure 6:**
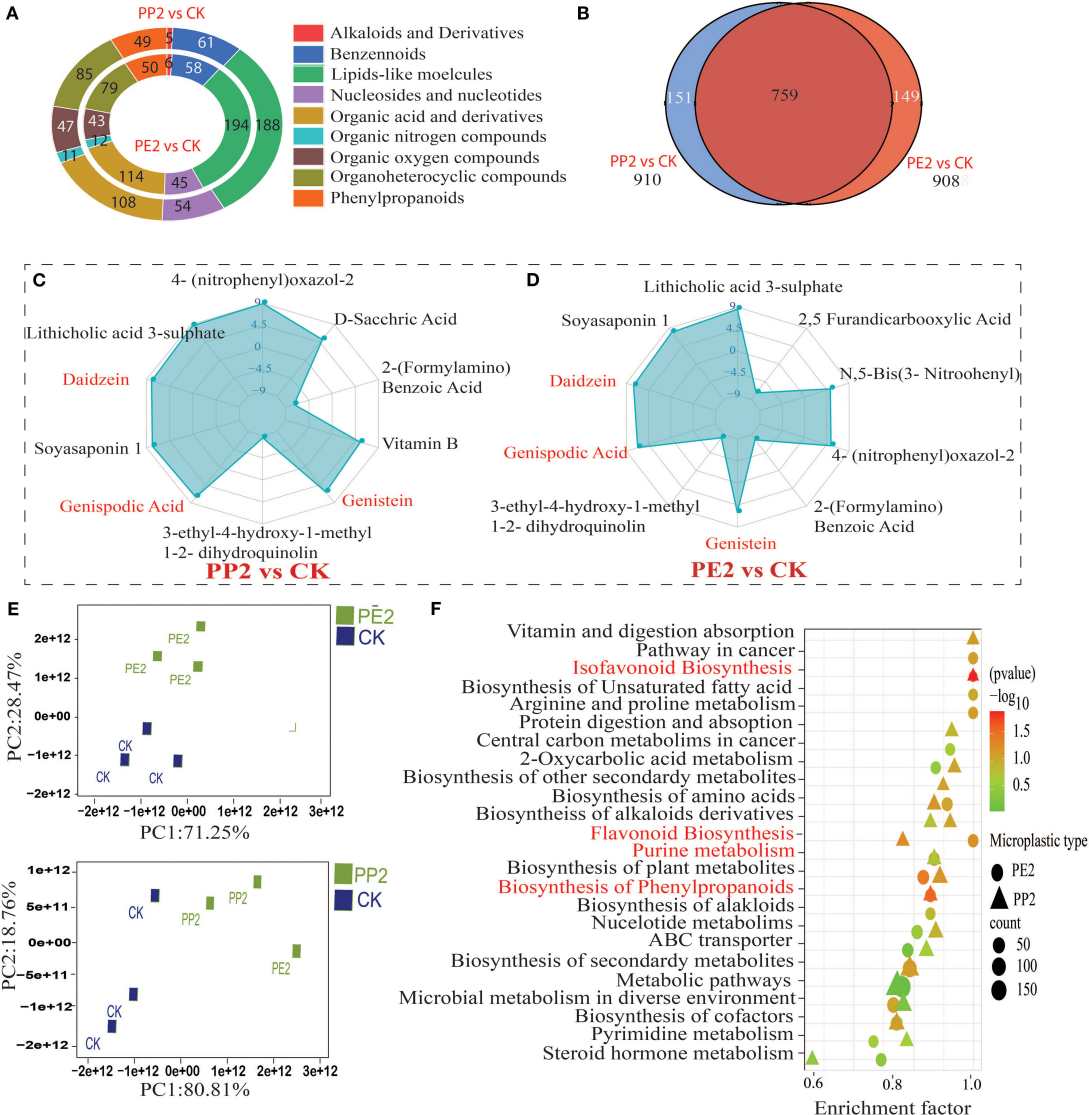
Effects of NPs on the metabolic profile of rhizosphere exudates and KEGG enrichment pathway analysis of differentially expressed metabolites (DEMs) are presented. Note: The selection criteria for DEMs were a VIP > 1 and P < 0.05. **(A)** The classification statistics and expression pattern analysis of DEMs in rhizosphere soil across different treatments; **(B)** Co-expression results of DEMs between treatments; **(C, D)** A Log2FC > 2 indicates significant upregulation and downregulation of metabolites; **(E)** Principal component analysis reveals differences in metabolic profile expression among the treatment groups **(F)** The enrichment results of the top 20 metabolic pathways are displayed in bubble charts;. Note: CK indicates control with no plastic particles added, PE2 represents the treatment with 500 mg/kg of polyethene, while PP2 and PP3 correspond to treatments with 500 mg/kg of polypropylene. All NPs were added exogenously to the soil.

We comprehensively analyzed metabolic profiles of the rhizosphere to understand changes in plant exudation under PP2 and PE2 treatments. UHPLC-MS/MS identified 908 DEM in the rhizosphere of soybean-planted soil under PP2 treatment, with 396 up-regulated and 512 down-regulated. Similarly, under PE2 treatment, 910 metabolites were detected, 47 up-regulated and 563 down-regulated. Lipid-like molecules were the most abundant in the metabolite composition, accounting for the largest share of total metabolites (**Figures 6A, B**). Compared to control, PE2 and PP2 treatments increased relative abundances of key metabolites, including geniposidic acid, Vitamin B2, Daidzein, Genistein, Naringenin, and phloretin, as well as lithocholic acid 3-sulfate, while reducing levels of Sulfamerazine, levodopa, L-, L-3-ethyl-4-hydroxy-1-methyl-1,2-dihydroquinolin-2-one (**Figures 6C, D**). PCA revealed a considerable divergence between treatments and control, underscoring the profound effect of NPs on rhizosphere metabolic deposition (**Figure 6E**). All DEM expressed were associated with approximately 20 metabolic pathways, according to KEGG enrichment analysis in NPs treatments, the most substantially enriched pathways (P < 0.01) were purine metabolism, phenylpropanoid biosynthesis, flavonoid biosynthesis and isoflavonoid biosynthesis (**Figure 6F**). Metabolomics data were derived solely from rhizosphere exudates, not directly from plant roots, which include both plant-derived exudates and bacterial products. While this approach may not fully capture the intricacies of plant signal transduction, careful sampling and detection procedures can minimize this effect. For the scope of this study, we focused on understanding how plant exudates influence the soil microbiome and symbiotic efficiency under NP exposure, with plans for further research to investigate plant-root interactions directly.

### 3.7 Correlation analysis of environmental factors, soil community structure, and metabolite interactions

**Figure 7:**
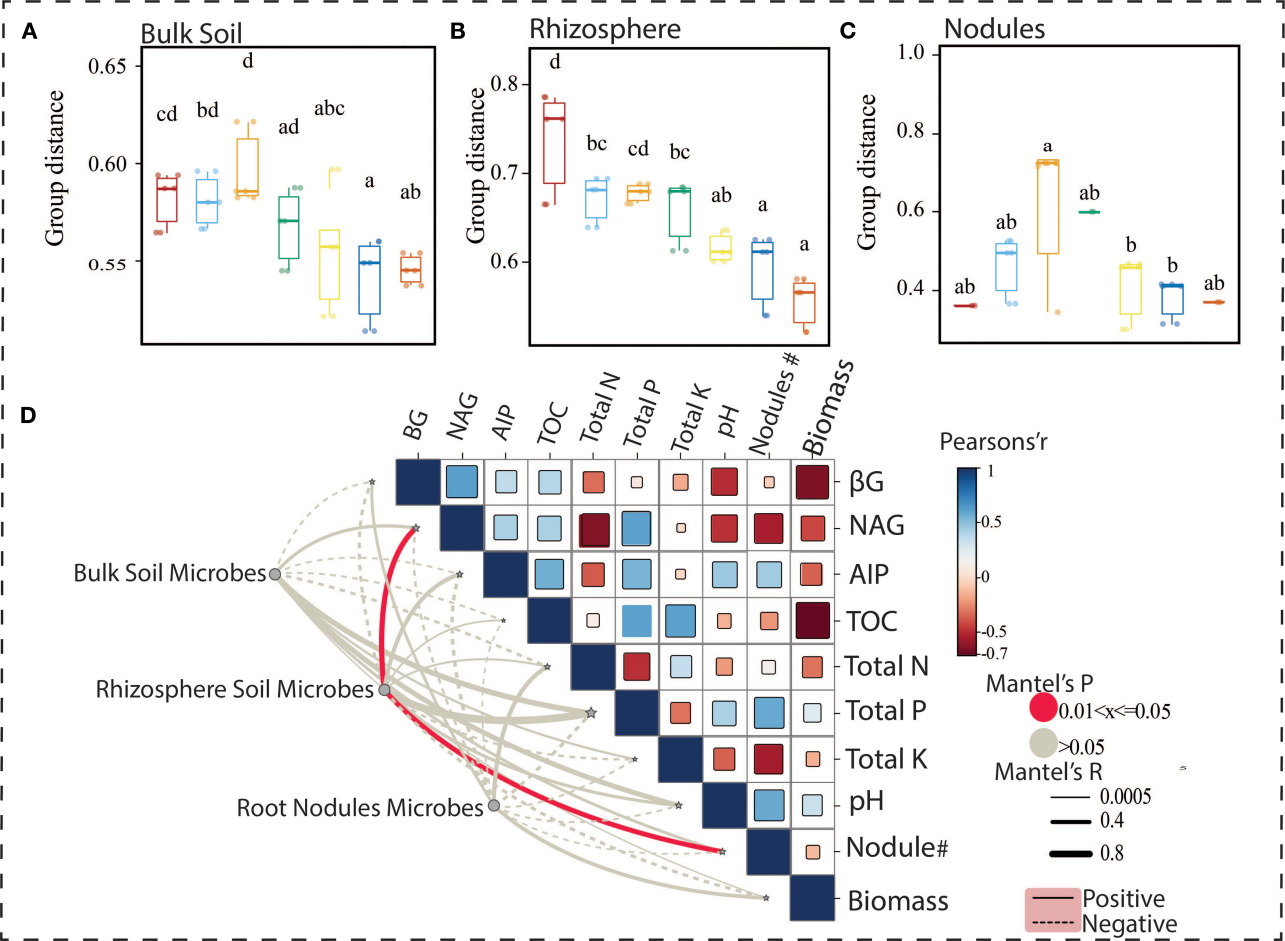
**(A–C)** The Jitter Box plot represents the unweighted UniFrac distance measured within treatments across different rhizocompartments. Values (mean ± SD, n = 5) with different superscripts differ significantly (Two-way ANOVA, Tukey’s HSD, p < 0.05). **(D)** The correlation matrix visualizes the relationships between several soil properties, including β-gluc (β-glucosidase activity), N-a (N-acetylglucosamine activity), AP (alkaline phosphatase activity), TOC (Total organic carbon), and Total N (Total nitrogen), with microbial groups in Bulk Soil, Rhizosphere, and Root Nodules. Pearson’s correlation coefficient (r) is shown, with colors indicating positive or negative correlations. The Mantel’s p-value is also displayed, with significance highlighted in red for p-values between 0.01 and 0.05 and non-significant values in blue.

Correlations were analyzed between the five most abundant genera of bulk soil, rhizosphere, and nodules and soil physicochemical properties, soil enzyme activities, and soybean growth indicators. The Mantel test revealed significant correlations between the bacterial community in the bulk soil (species B) and the total nitrogen content (P < 0.05). Similarly, the microbial community in the rhizosphere (species R) was significantly correlated with the number of plant nodules (P < 0.05). A notable correlation was also observed between the nodule’s microbial community and the alkaline phosphatase activity in the soil (P < 0.05). Pearson’s correlation analysis also showed that soil TP and AIP activity were positively correlated with soil pH, soil TOC content, and NAG activity with soybean nodulation. On the contrary, NAG was negatively correlated with total nitrogen, nodules, and biomass of the plant (**Figure 7**). These findings suggest that the bacterial communities in different rhizocompartments are influenced by different soil factors, like PH, TN, TC, and TP, which are particularly critical in determining plant biomass and nodulation.

In the bulk soil under PE2 treatment, the microbial and metabolic profile co-occurrence network comprised 71 nodes and 46 edges, resulting in an average degree of 1.29 and 80.43% positive links, indicating a highly positive interaction network. On the contrary, the PP2 treatment produced a smaller network with 43 nodes and 23 edges, a lower average degree of 1.07, and only 30.43% positive links. The PE2 treatment exhibited a similar pattern in the rhizosphere, with 15 nodes and 10 edges, an average degree of 1.33, and 80% positive links. However, the PP2 treatment resulted in a smaller network of 12 nodes and 6 edges, with just 50% positive links. In the nodules, the PE2 treatment produced a network of 57 nodes and 39 edges, with an average degree of 1.36 and 69.23% positive links, while the PP2 treatment produced a smaller network of 52 nodes and 28 edges, with 57% positive links. Overall, the data reveal positive correlations outweighed negative ones, with PE2 exposure leading to a more complex co-occurrence network than PP2 treatment. In particular, (2R)-2-[(2R,5S)5[(2S)2-hydroxybutyl]oxolan-2-yl] propanoic acid emerged as the most commonly co-occurring metabolite under PE2 in all compartments. Under PE2 and PP2, liquiritigenin, daidzein, genistein, cis-tonic acid, and L-glutamine in the rhizosphere and nodules were prominently associated with bacterial genera such as *Bradyrhizobium, Ensifer, and Pedobacter* (**Supplementary Figure S4**).

## 4 Discussion

### 4.1 NPs stimulated nodulation and biological fixation of N

Our findings reveal that NPs treatments induced higher nodulation and increased nitrogenase activity in nodules, while also resulting in a general reduction in the plant nitrogen level. The decline in nitrogen content in legumes may indicate an improvement in symbiotic N_2_ fixation (Gubsch et al., 2011). Similarly, previous studies have reported that nanosized plastics induced nitrogen deficiency, leading to higher nodulation in peanuts (Wang et al., 2023). According to our results, NPs exposure induced changes in various forms of nitrogen in both soil and plants, which supports the hypothesis that plastics may induce nitrogen deficiency in legume plants. However, at lower doses of NPs, the soil NH_4_^+^ levels remained relatively stable, likely due to increased nodulation, the primary outcome of biological nitrogen fixation. The mechanism behind the effects of NPs treatments on plant growth and nodulation appears to be related to changes in soil physicochemical properties, altered plant exudation in the rhizosphere, and changes in microbial composition and assembly processes in different ecosystems.

### 4.2 Response of soil physicochemical variables to NPs

Both polymers caused a notable decrease in soil pH overall, with higher doses of plastics leading to a more pronounced effect. This decrease could be attributed to altered cation exchange capacity, as previous studies have shown that PE plastics reduce soil pH and affect cation exchange capacity (Wang et al., 2021). Under planted conditions, NPs can influence the release of root exudates, including H^+^ ions and low molecular weight organic acids, which can affect soil pH (Dong et al., 2022). Undoubtedly, plastic contamination in the soil could lead to an increase in organic and total carbon levels, as NPs are carbon-containing polymers. However, NPs differ from typical soil particles because most microorganisms do not easily degrade them (Rillig, 2018). Our results showed a significant increase in TOC availability, except at low doses of PP, which aligns with previous reports (Ma et al., 2024; Shoaib et al., 2025). In general, the impact of NPs on soil carbon can vary, as they may increase it (Feng et al., 2022), decrease it (Meng et al., 2022), or have an insignificant effect (Li et al., 2021). These effects are highly dependent on the initial properties of the soil, as well as the type and duration of exposure to NPs. With low aromaticity, PP plastics may preferentially adsorb nonaromatic dissolved organic carbon compounds, potentially disrupting soil carbon dynamics and altering the overall carbon cycling process (Li et al., 2021). In our study, soil P availability and related enzyme activities were strongly correlated with soil carbon, nitrogen cycling enzymes, and soil pH. Acid phosphatase (AcP) and alkaline phosphatase (AlP) are adapted to specific pH ranges, making soil pH a key factor in regulating P-related enzyme activity (Shujie et al., 2021). According to previous reports, AcP and AlP activities are correlated with soil pH, total carbon, and total nitrogen (Li et al., 2021). A plausible explanation for the strong connections between P-related enzyme activity, soil carbon, and nitrogen-cycling enzymes is that soil microbes produce these enzymes to obtain limited nutrients, which helps maintain the balance of elements (Vitousek et al., 2010). NAG activity typically correlates with increased nitrogen mineralization rates, indicating a positive relationship between NAG activity and soil nitrogen availability (Geisseler and Horwath, 2009). However, in the presence of NPs, NAG activity has been negatively correlated with nitrogen content, suggesting that while increased enzymatic activity may initially enhance nitrogen mineralization, it ultimately impairs nitrogen cycling. This shift in nitrogen dynamics could improve nodulation, as nitrogen availability is essential for the formation and function of root nodules in legumes. In our study, the differential effects of PE and PP-NPs on soil enzyme activities stem from their distinct physicochemical properties. PE, with a lower density and greater flexibility, increases its surface area and enhances interactions with soil microbes and enzymes (Wang et al., 2021). In contrast, PP is more rigid and hydrophobic, which makes it less prone to microbial colonization and enzymatic degradation, resulting in a weaker impact on soil biochemical processes (Li et al., 2024).

### 4.3 Responses of the diversity and composition of the soil microbial community

NPs, as widespread anthropogenic pollutants, exert evolutionary pressure on soil microorganisms, especially those with short generation times, reshaping their ecological dynamics (Cregger et al., 2021). In our study, the α-diversity indices remained unchanged or increased with the addition of NP, except at higher dosages of PP NPs, where a decrease was observed. The bacterial composition changed significantly under NPs treatments, resulting in notable alterations in the abundance of specific microbial taxa. Although NPs did not decrease the overall number of microbial species, they may have affected the abundance of specific microbial groups. NPs serve as artificial surfaces for microbial attachment, facilitating colonization and contributing to shifts in community dynamics (Wang et al., 2018).

Furthermore, high concentrations of NPs exert selection pressure on soil microbes, altering the structure and diversity of the microbial community, with potential evolutionary consequences (Paramdeep et al., 2022). We detected a surge in the relative abundance of *Acidobacteriota, Actinobacteria, and Proteobacteria* in bulk and rhizosphere soils. This pattern aligns closely with (Zhang et al., 2020), who identified *proteobacteria and actinobacteria* as key microbial players in the degradation of field-collected plastic mulch. These bacteria are also well documented as pioneering colonizers of artificial surfaces in environmental settings, highlighting their role in plastic degradation (Rampelotto et al., 2013). In our study, most bacteria that thrive under NPs treatments belong to the *Actinobacteria* phylum, including genera such as *Streptomyces*, *Nocardia*, and *Arthrobacter.* Previous studies have shown that species such as *Streptomyces* can biodegrade polyethylene by producing hydrolytic enzymes (Abraham et al., 2017). Additionally, *Arthrobacter* has been identified as a potent degrader of nonbiodegradable and persistent compounds, underscoring the ecological significance of these microbes in mitigating plastic pollution, particularly NPs (Goel et al., 2008).

### 4.4 NPs-induced shifts in bacterial community assembly and nodule symbiosis dynamics

The symbiotic process begins in the rhizosphere, where bacteria are recruited and selected after bacterial infection of root hairs. In the control treatment, we observed a slight increase in deterministic processes (i.e., processes driven by specific environmental factors) as we moved from the bulk soil to the nodule. The notable impact of plastic treatments on the composition of the bacterial population in each compartment suggests that NPs altered the bacterial assembly process across the bulk soil-rhizosphere-nodule ecosystem. We observed a growing trend in the impact of NPs on the bacteriome, which transitions from bulk soil to nodules, indicating a cascade of microbial shifts that amplify the impact of NPs on the homeostasis of the nodule bacteriome and plant symbiotic potential, as discussed below. Under NPs treatments, we observed a slight increase in species exchange between the bulk soil and rhizosphere (**Figure 4**), indicating that low concentrations of nanosized plastics may promote species dispersal. This finding is further supported by the relatively lower DL observed in the bulk soil and rhizosphere with NPs treatments. However, the impact of NPs on community assembly in these compartments was relatively modest, as the proportions of selection and stochastic processes (DL and drift) remained stable. In our results, NPs treatments substantially influenced the community assembly mechanisms in nodules. The assembly of the nodule community was highly driven by HoS under PE2 and PP2 treatments, compared to the rhizosphere, suggesting that the nodule bacterial community experienced intense selection pressures. This is logical because nodules provide a niche for capturing symbiotic diazotrophs like *Bradyrhizobium*, as illustrated in the bar plots (**Figure 3**). Nodules are specialized organs that contain high levels of plant-fixed carbon and low oxygen concentrations, making them essential for biological nitrogen fixation (Gautrat et al., 2021). NPs treatments increased the diversity of assembly of the nodule bacterial community, as evidenced by the higher DR observed under these treatments (**Figure 5**). These treatments also supported lower turnover and increased stochasticity, probably due to NPs facilitating greater species enrichment in the nodule from the rhizosphere. With NPs exposure, species such as *Streptomyces*, *Nocardia*, and *Arthrobacter* were more enriched in the nodule compared to the rhizosphere. These species may enhance nodulation by interacting positively with the symbiotic diazotroph *Bradyrhizobium*. Certain species of *Streptomyces* have also been reported to promote soybean nodulation by *Bradyrhizobium* (Gregor et al., 2003; Méndez-Camarillo et al., 2024). The alterations in the assembly of the bacteriome under NPs treatments can be partly explained by the formation of a plastisphere, which introduces a new level of complexity. The plastisphere applies selective pressure on the bacteriome when it attaches to the root surface. Notably, *γ-Proteobacteria*, *Actinobacteria*, and *Bacteroidetes* are often enriched in the plastisphere of PE, which influences the microbial dynamics in the root and nodule environment (Wang et al., 2022). Symplastic and apoplastic routes allow NPs to penetrate the root, further affecting the dynamics of microbial populations (Bansal et al., 2024; Rong et al., 2024). Our results indicate that the NPs-mediated plastisphere significantly contributes to the nodule microbiome, with a greater impact than micron-sized plastics, as highlighted in previous studies (Liu et al., 2024; Luo et al., 2025). Furthermore, NPs can affect the plant root metabolome and the allocation of photosynthetic carbon, thus influencing the assembly of microbial communities in both the roots and nodules (Chen et al., 2022; Hu et al., 2024), a central focus of our study.

### 4.5 NPs exposure influences rhizobial proliferation

Successful symbiosis between rhizobia and their legume hosts is co-regulated, with nodulation rates varying based on environmental factors and the specific rhizobia involved. *Bradyrhizobium* and *Ensifer* are soybeans’ primary microsymbionts, each exhibiting different nodulation capabilities. These rhizobia also participate in competitive interactions in soil, varying their performance under different pH levels (Rodríguez-Navarro et al., 2011). Our study found that low doses of NPs accelerated *Bradyrhizobium* proliferation, which is consistent with previous research linking NPs to biomarker taxa involved in nitrogen cycling in Biomedical Polymers-treated sediment (Wang et al., 2023). Our work highlights the key role of *Ensifer* in enhancing nodulation capacity under exposure to NPs. *Ensifer* species are known for their resilience under harsh environmental conditions. They thrive by producing osmoprotectants such as trehalose and glycine betaine, which help them survive under stress (Belfquih et al., 2021). For example, *Ensifer medical MA11* has been shown to effectively enhance the symbiotic potential of *Medicago* species under arsenic stress, highlighting its potential as a bioinoculant for contaminated soils (Lafuente et al., 2015). These findings emphasize the critical roles of *Bradyrhizobium* and *Ensifer* in enhancing nodulation, even under NPs-induced stress.

### 4.6 NPs mediated flavonoid and isoflavonoid biosynthesis favors the symbiotic process

The uptake of NPs by plants is well documented. Studies have shown that NPs ranging in size from nanometers to micrometers can be absorbed by *Arabidopsis thaliana*, *Triticum aestivum* (wheat), and *Lactuca sativa* (lettuce) through crack entry mechanisms as early as seven days after sowing. This leads to tissue accumulation and subsequent physiological disruptions (Li et al., 2020; Ren et al., 2024; Sun et al., 2020). In the present study, soybean roots showed higher accumulation of PE-NPs compared to PP-NPs. This may be due to the higher hydrophobicity and slightly greater surface energy of PE-NPs, enhancing their adhesion and retention on root surfaces (Hadiyanto et al., 2021). NPs accumulation exacerbated the toxic effects, leading to increased membrane lipid damage and activation of the antioxidant response in soybean roots, consistent with previous findings (Surgun-Acar, 2022). However, this aspect is beyond the scope of our study. Our results are consistent with previous studies, NPs exposure at low doses can lead to changes in the quantity and composition of plant root exudates, which in turn interact with and influence soil microbes. However, NPs long-term exposure impaired plant growth and development (Liu et al., 2024). According to our results, under NPs exposure, soybean triggered the biosynthesis of flavonoids and isoflavonoids. It can be concluded that plants grown with NPs prioritize the protection of their symbiosis and nitrogen fixation by enhancing the synthesis of these compounds, at the expense of above-ground biomass.

Flavonoids and isoflavonoids serve as a chemoattractant for rhizobia species during nodule development (Mapope and Dakora, 2012). These phenolic compounds facilitate rhizobia chemotaxis, promote bacterial proliferation, and activate nodulation (nod) gene expression in compatible strains (Mandal et al., 2010). In this study, we observed significant up-regulation of metabolites such as naringenin, phloretin, kaempferol (flavonoid pathway), and daidzein, coumestrol, genistein (isoflavonoid pathway) as the most expressed DEMs in both NPs exposure types, consistent with previous studies showing altered gene expression and metabolism in these pathways in peanut plants (Wu et al., 2024). Naringenin and phloretin are key flavonoids that enhance plant-microbe interactions under stress, promote nutrient uptake, and enhance the plant defense mechanism (Shah and Smith, 2020). Daidzein, coumestrol, and genistein are crucial for legumes like soybeans, promoting nodulation, enhancing nitrogen fixation, and supporting stable crop yields under stress (Okutani et al., 2020). Similar to our findings, previous studies show that CeO_2_ nanoparticle exposure induces stress in soybean plants, altering physiology and exudate profiles, which in turn affect root-associated microbial communities (Reichman et al., 2024). Our findings are consistent with this mechanism, with the added observation that NPs in the soil directly promoted rhizobial proliferation and influenced microbial assembly processes. Indirectly, the uptake of NPs by soybeans likely alters their physiology and exudate composition, particularly flavonoids, favoring symbiotic interactions. Both direct and indirect NPs effects mediated changes appeared to be the dominant driver of microbial shifts in our study.

## 5 Research limitations and future perspectives

While this study offers valuable insights into the effects of NPs exposure on plant-microbe interactions, a few limitations warrant consideration. Firstly, the physicochemical properties of NPs were not included in the current analysis. Given the substantial impact of soil ionic strength on the physicochemical properties of NPs, future studies must incorporate to assess both the size distribution and aggregation state of NPs in the soil solution. Additionally, Longitudinal data collection of soil enzyme activities and nutrient concentrations at multiple time points would facilitate a deeper understanding of the temporal dynamics of microbe-plant interactions and the progressive effects of NPs on soil biochemical processes. Furthermore, while community composition analysis provided valuable insights into microbial shifts, the validation of key functional genera, such as nitrogen-fixing *Bradyrhizobium*, through techniques like qPCR, would offer more robust evidence of functional gene expression changes, particularly those related to nitrogen fixation, such as the *nifH* gene. Despite these limitations, the findings of this study provide a foundational framework for future research on the effects of NPs on soil-plant-microbe interactions. By addressing these critical data gaps, future studies will improve our understanding of NPs bioavailability, as well as their broader ecological implications and potential risks.

## 6 Conclusions

In this study, we investigated the impact of two different types of NPs (PE and PP) with varying concentration levels on the soil’s physicochemical properties, microbial community composition, assembly, and symbiotic performance in soybean. Our findings revealed that the effects of these materials on soil properties depended on the type of polymer, with PE significantly enhancing soil enzymatic activities. NPs alter the delicate balance between N-acetyl-β-D-glycosaminidase (NAG) activity and nitrogen transformation in the soil, ultimately disrupting nutrient cycling. NPs have been shown to promote nodulation and biological nitrogen fixation in legume plants, while also influencing the homeostasis of soil bacteriomes. NPs can enrich bacterial genera associated with the nitrogen cycle and potentially enhance the symbiotic potential of plants, although the effects may vary depending on the concentration of NPs. However, changes in the assembly of the bacterial community in bulk soil, rhizosphere, and nodule ecosystems due to soil NPs pollution may alter plant-microbe symbiosis and biological nitrogen fixation, driven by microbial flow in different niche compartments. NPs induced plant rhizosphere exudation, particularly the biosynthesis of flavonoids and isoflavonoids, as well as their metabolites, such as genistein, naringin, and daidzein, which support plant symbiotic processes. These findings significantly improve our understanding of the impact of NPs on soil microbial composition and assembly, as well as their ultimate effect on plant-microbe interactions for successful symbiosis and nitrogen fixation.

## Data Availability

The datasets presented in this study can be found in online repositories. The names of the repository/repositories and accession number(s) can be found below: https://www.ncbi.nlm.nih.gov/, PRJNA1156063, PRJNA1156377, and PRJNA1156528.
