# Supplementary material for: Nanoplastic alters soybean microbiome across rhizocompartments level and symbiosis via flavonoid-mediated pathways
Source: Front Plant Sci. 2025 Oct 2;16:1676933. doi: 10.3389/fpls.2025.1676933 (PMC12528142; doi:10.3389/fpls.2025.1676933)
Supplement: Supplementary file 1 [file DataSheet1.docx]

**Nanoplastic Alter Soybean Microbiome Across Rhizocompartments Level and Symbiosis via Flavonoid-Mediated Pathways**

**1. Supplementary Material**

- 1. **Materials and methods**
  2. **Physicochemical properties of the tested soil and the method used for testing**

"The tested soil was classified as a sandy loam with the following properties (mean ± SD, n=3):

pH: 6.8 ± 0.2 (in H₂O, 1:2.5 w/v)

Organic matter: 2.1 ± 0.3% (loss-on-ignition method)

Cation exchange capacity (CEC): 12.5 ± 1.5 cmol₊/kg

Total nitrogen: 0.15 ± 0.02% (Kjeldahl digestion)

Available phosphorus (Olsen P): 11.3 ± 2.1 mg/kg (NaHCO₃ extraction)

Electrical conductivity (EC): 0.8 ± 0.1 dS/m (1:5 soil: water extract).

**1.3 Morphological characteristics of nanoplastic**


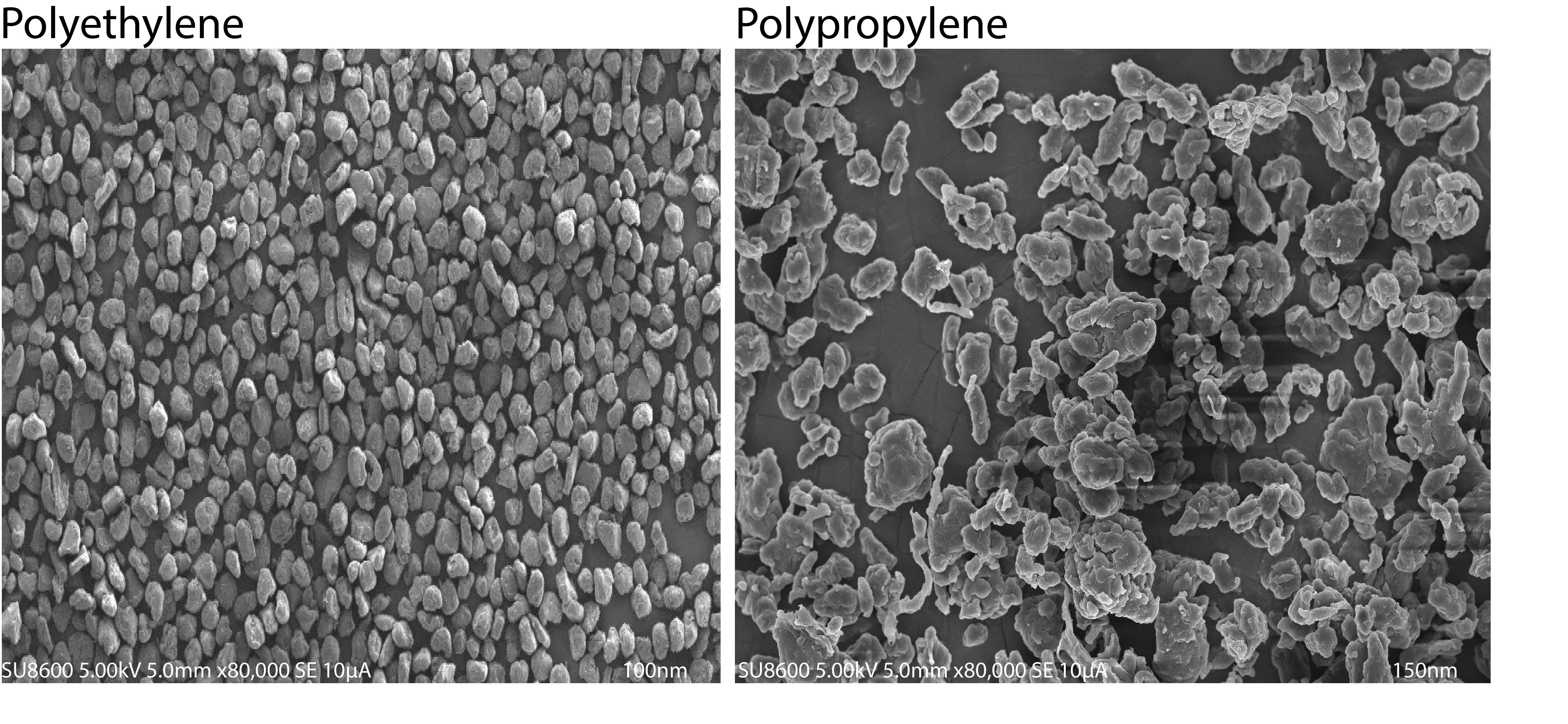


The morphological characteristics of the Polyethylene and Polypropylene nanoplastics (20 nm to 50nm) were examined using scanning electron microscopy (SEM) (HITACHI SU8600 and JW-BK200C, Gaobo, China).

**1.4.** **Soil physicochemical analysis**

We measured soil pH using a pH meter with a 1:2.5 (w/w) soil-to-water ratio (Kerley et al., 2001). Total phosphorus in the soil was extracted using solutions of 0.5 M NaHCO₃ and 0.01 M CaCl₂, and its concentration was analyzed using the molybdenum blue colourimetric method (Daly et al., 2005). The potassium content of the soil was measured with a 1 M ammonium acetate solution, and its concentration was measured by flame photometry (Lu et al., 2017). The total nitrogen content was determined using the Kjeldahl method, followed by the digestion of soil samples with sulfuric acid (Hasibuan et al., 2015). Soil NO_3_^–^ and NH_4_^+^ were extracted using KCl solution (2 M) and determined using UV spectroscopy as previously described (Wheatley et al., 1989). The total carbon content was measured using a TOC analyzer (Vario TOC, Elementar Analyser System GmbH, Germany) in a 1:3 (w/v) soil-to-water suspension, following the method described by (Kalbitz et al., 2003).

For soil bacterial enzyme activities, we assessed β-1,4-glucosidase (BG), N-acetyl-β-glycosaminidase (NAG), and alkaline phosphatase (AlP). BG plays a key role in cellulose degradation by hydrolyzing cellobiose to glucose (Hendriksen et al., 2016). NAG is responsible for hydrolyzing chitin chains and releasing N-acetylglucosamine (Dick et al., 2011), while AlP catalyzes the hydrolysis of organic ester phosphorus to phosphate (Nannipieri et al., 2011). BG is critical for soil carbon cycling, and NAG is often used as an indicator for soil nitrogen cycling (Lin et al., 2020; Tian et al., 2022). AlP is a key enzyme in soil phosphorus cycling (Hu et al., 2022). Soil enzymatic activities were determined using a high-throughput microplate assay (Jackson et al., 2013). For enzyme activity measurement, we used model substrates linked to p-nitrophenyl (pNP): pNP-D-glucopyranoside (Sigma no. N7006), pNP-N-acetyl-D-glucosaminide (Sigma no. N9376), and pNP phosphate disodium salt hexahydrate (Sigma no. 71,768). A total of 3.5 g of frozen soil from each sample (stored at −20 °C) was placed in a 50 ml sterile centrifuge tube and mixed with 10 ml of 50 mM acetate buffer. The mixture was vortexed for 30 seconds to create a soil slurry. After centrifugation, the soil slurry was transferred to 96-well microplates, with each well containing 0.150 ml of the soil slurry and 0.150 ml of substrate dissolved in 50 mM sodium acetate buffer (pH 5.0). The reaction mixtures were then incubated at 25 ° C for 2 to 4 hours. Following incubation, the mixtures were centrifuged at 3,000 rpm for 5 minutes and 0.100 ml of suspension was transferred to a new microplate. To stop the reaction, 0.200 ml of 0.1 M NaOH was added. The absorbance was measured spectrophotometrically at 410 nm using a microplate reader (BioRad, Benchmark Plus, Japan) (Xu et al., 2024).

**1.5. Measurement of NPs accumulation in roots**

Measurement of NPs concentration in soybean roots began with surface cleaning of the root tissue, followed by drying in an oven at 60 °C until a constant weight was reached. The dried roots were then ground into a fine powder. Afterwards, 30 mL of a 30 % H₂O₂ solution was added to the beaker, which was then placed in a gas bath at 50°C and shaken continuously for 24 h to digest the plant matter. After digestion, the mixture was filtered under vacuum using a [glass fiber](https://www.sciencedirect.com/topics/pharmacology-toxicology-and-pharmaceutical-science/glass-fiber) filter membrane with the smallest available pore size. The filter membrane was then dried and stored for further processing. To extract the NPs, the dried membrane was immersed in a mixed [organic solvent](https://www.sciencedirect.com/topics/biochemistry-genetics-and-molecular-biology/organic-solvents) and subjected to [ultrasonic treatment](https://www.sciencedirect.com/topics/agricultural-and-biological-sciences/ultrasonic-treatment) to dissolve or detach plastic particles. The resulting solution was concentrated to 50–100 µL for analysis. A glass pipette was used to place droplets of the solution into a pyrolysis-GC/MS (Py-GC/MS) crucible. After complete solvent evaporation, the samples were analyzed. Calibration standards of known NPs concentrations were also prepared, and Py-GC/MS was used to construct a quantitative standard curve ([Rauert et al., 2025](https://www.sciencedirect.com/science/article/pii/S0147651325009571#bib44)).

**1.6 Antioxidant response index measurement in soybean root**

Root samples were collected from five independent plants per treatment group (biological replicates. The protocol by Cakmak et al., (1991) was used to assess malondialdehyde (MDA). A 0.25 g fresh root sample was homogenized in 2 mL of 0.1% (w/v) tricarboxylic acid (TCA) and centrifuged at 1500 rpm for 20 minutes. For MDA estimation, 1 mL of the supernatant was mixed with 4 mL of thiobarbituric acid (TBA) reagent, prepared by dissolving 0.5 g TBA and 20 g TCA in 100 mL of distilled water. The mixture was incubated at 95 °C for 30 minutes and allowed to cool. Absorbance was measured at 532 nm and 600 nm using a spectrophotometer. Superoxide dismutase (SOD) activity was measured following the method of Giannopolitis et al., (2016), using a reaction mixture containing: 50 µL enzyme extract, 50 µL riboflavin, 50 µL nitroblue tetrazolium (NBT), 250 µL phosphate buffer, 100 µL methionine, 100 µL Triton X-100, and 40 µL distilled water. The mixture was exposed to light for 20 minutes, and absorbance was measured at 560 nm. Superoxide dismutase (SOD) activity was measured following the method of Giannopolitis and Ries, using a reaction mixture containing: 50 µL enzyme extract, 50 µL riboflavin, 50 µL nitroblue tetrazolium (NBT), 250 µL phosphate buffer, 100 µL methionine, 100 µL Triton X-100, and 40 µL distilled water. The mixture was exposed to light for 20 minutes, and absorbance was measured at 560 nm. **Catalase (CAT)** activity was determined following (Aqeel et al., 2021). The assay mixture included 100 µL enzyme extract, 1.9 mL phosphate buffer, and 1 mL of 5.9 mM H₂O₂ in distilled water. Absorbance was recorded at 240 nm every 20 seconds using a spectrophotometer.

**Supplementary Figures**

**Figure S1:** Phenotypic assessment of soybean and nodulation under different nanoplastic (NPs) treatments. Five biological replicates were grown for each treatment; however, only three representative plants per treatment were selected for photographic presentation to ensure clear visual comparison.





**Figure S2**: Nanoplastic accumulation in roots **(a)**, Changes in **(**MDA**)** content **(b)**, SOD **(c)**, and CAT activity **(d)** of soybean roots. Note: CK indicates control with no plastic particles added, and PE1, PE2, and PE3 represent the treatments with 200, 500, and 1000 mg/kg of polyethene, respectively. While PP1, PP2, and PP3 correspond to treatments with 200, 500, and 1000 mg/kg of polypropylene. All nanoplastics were added exogenously to the soil. Values (mean ± SD, n = 5) with different asterisks (***,**,* and letters a,b,c) differ significantly (Two-way ANOVA, Tukey's HSD, p < 0.05).


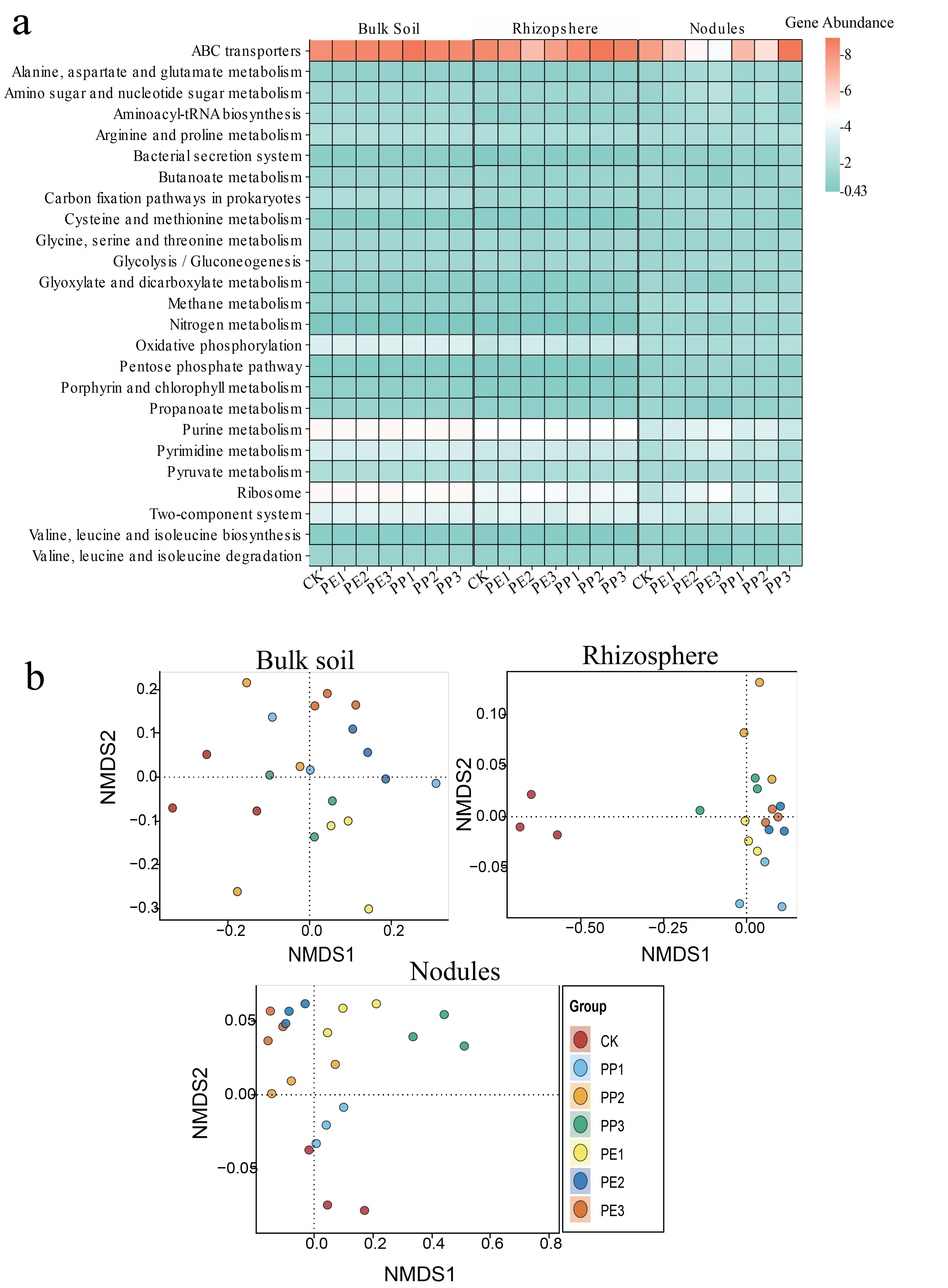


**Figure S3:** **(a)** Prediction results of KEGG functions based on 16S rRNA sequences (Top 20**)**, **(b)** NMDs (non-metric multidimensional scaling (NMDS) analysis of microbial communities at rhizocompartments levels: Bulk soil, Rhizosphere, and Nodules. Note: CK indicates control with no plastic particles added, and PE1, PE2 and PE3 represents the treatments with 200, 500 and 1000 mg/kg of polyethene, respectively. While PP1, PP2 and PP3 correspond to treatments with 200, 500 and 1000 mg/kg of polypropylene. All NPs were exogenously applied to the soil.


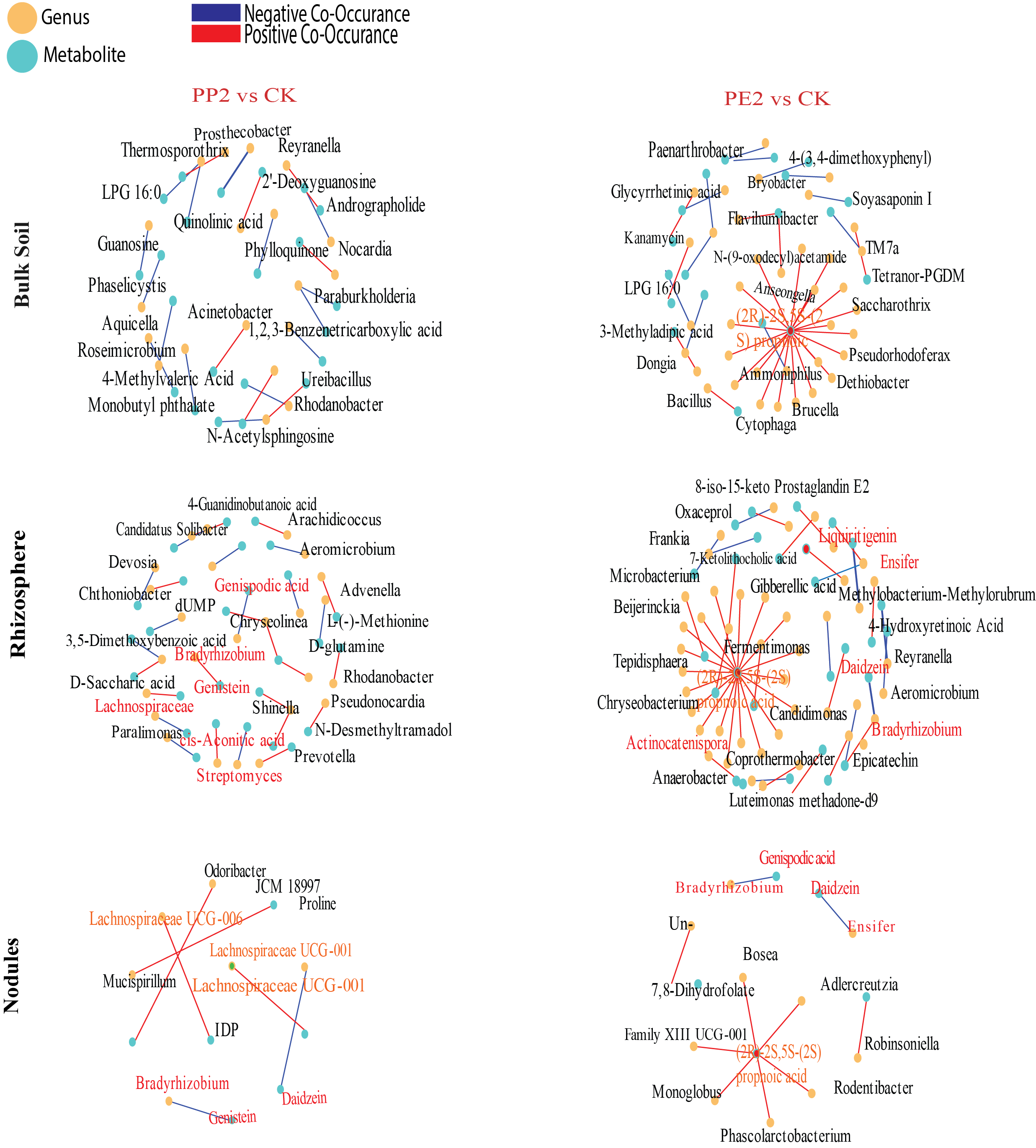


**Figure S4:** The interaction network between the soil microbial community at the genus level and differentially expressed plant metabolites (DEMs ) was mapped across different rhizocompartments levels, with a correlation coefficient > 0.5 and P<0.05. The blue line represents negative correlation, and the red line represents positive correlation. Red colour represented the most correlated DEMs with different microorganisms. The CK showed that no particles were added, PE2 showed that 500mg/kg of polyethylene, and PP2 showed 500mg/kg of polypropylene, following the exogenous application of these NPs in soil.

**Referencs**

Aqeel, M., Khalid, N., Tufail, A., Ahmad, R. Z., Akhter, M. S., Luqman, M., Javed, M. T., Irshad, M. K., Alamri, S., & Hashem, M. (2021). Elucidating the distinct interactive impact of cadmium and nickel on growth, photosynthesis, metal-homeostasis, and yield responses of mung bean (Vigna radiata L.) varieties. *Environmental Science and Pollution Research*, *28*, 27376-27390.

Bates, L. S., Waldren, R., & Teare, I. (1973). Rapid determination of free proline for water-stress studies. *Plant and soil*, *39*, 205-207.

Cakmak, I., & Horst, W. J. (1991). Effect of aluminium on lipid peroxidation, superoxide dismutase, catalase, and peroxidase activities in root tips of soybean (Glycine max). *Physiologia plantarum*, *83*(3), 463-468.

Daly, K., & Casey, A. (2005). Environmental aspects of soil phosphorus testing. *Irish journal of agricultural and food research*, 261-279.

Dick, R. P. (2011). Methods of soil enzymology.

Hasibuan, M. E. (2015). *Analisa Kadar Nitrogen pada Pupuk Urea dengan Metode Kjeldhal di PT Sucofindo Medan* Universitas Sumatera Utara].

Hendriksen, N., Creamer, R., Stone, D., & Winding, A. (2016). Soil exo-enzyme activities across Europe—The influence of climate, land-use and soil properties. *Applied Soil Ecology*, *97*, 44-48.

Hu, Y., Chen, J., Hui, D., Wang, Y. P., Li, J., Chen, J., Chen, G., Zhu, Y., Zhang, L., & Zhang, D. (2022). Mycorrhizal fungi alleviate acidification‐induced phosphorus limitation: Evidence from a decade‐long field experiment of simulated acid deposition in a tropical forest in south China. *Global Change Biology*, *28*(11), 3605-3619.

Jackson, C. R., Tyler, H. L., & Millar, J. J. (2013). Determination of microbial extracellular enzyme activity in waters, soils, and sediments using high throughput microplate assays. *JoVE (Journal of Visualized Experiments)*(80), e50399.

Kalbitz, K., Schmerwitz, J., Schwesig, D., & Matzner, E. (2003). Biodegradation of soil-derived dissolved organic matter as related to its properties. *Geoderma*, *113*(3-4), 273-291.

Kerley, S., Shield, I., & Huyghe, C. (2001). Specific and genotypic variation in the nutrient content of lupin species in soils of neutral and alkaline pH. *Australian journal of agricultural research*, *52*(1), 93-102.

Lin, D., Yang, G., Dou, P., Qian, S., Zhao, L., Yang, Y., & Fanin, N. (2020). Microplastics negatively affect soil fauna but stimulate microbial activity: insights from a field-based microplastic addition experiment. *Proceedings of the Royal Society B*, *287*(1934), 20201268.

Lu, D., Li, C., Sokolwski, E., Magen, H., Chen, X., Wang, H., & Zhou, J. (2017). Crop yield and soil available potassium changes as affected by potassium rate in rice–wheat systems. *Field Crops Research*, *214*, 38-44.

Nannipieri, P., Landi, L., Giagnoni, L., & Renella, G. (2011). Past, present and future in soil enzymology. In *Soil Enzymology in the Recycling of Organic Wastes and Environmental Restoration* (pp. 1-17). Springer.

Sharma, A., Wang, J., Xu, D., Tao, S., Chong, S., Yan, D., Li, Z., Yuan, H., & Zheng, B. (2020). Melatonin regulates the functional components of photosynthesis, antioxidant system, gene expression, and metabolic pathways to induce drought resistance in grafted Carya cathayensis plants. *Science of The Total Environment*, *713*, 136675.

Tian, J., Wei, K., Sun, T., Jiang, N., Chen, Z., Feng, J., Cai, K., & Chen, L. (2022). Different forms of nitrogen deposition show variable effects on soil organic nitrogen turnover in a temperate forest. *Applied Soil Ecology*, *169*, 104212.

Wheatley, R., MacDonald, R., & Smith, A. M. (1989). Extraction of nitrogen from soils. *Biology and Fertility of Soils*, *8*, 189-190.

Xu, Q., Wang, X., Wang, N., Li, S., Yao, X., Kuang, H., Qiu, Z., Ke, D., Yang, W., & Guan, Y. (2024). Nitrogen inhibition of nitrogenase activity involves the modulation of cytosolic invertase in soybean nodule. *Journal of Genetics and Genomics*, *51*(12), 1404-1412.
